# Clinical and microbiological characterization of *Aerococcus urinae* bacteraemias at Helsinki metropolitan area, Finland

**DOI:** 10.1007/s10096-022-04415-6

**Published:** 2022-03-08

**Authors:** Reetta Sihvonen, Maria Turunen, Laura Lehtola, Laura Pakarinen, Juha O. Grönroos, Kaisu Rantakokko-Jalava, Anu Pätäri-Sampo

**Affiliations:** 1 Department of Clinical Microbiology, HUSLAB, University of Helsinki and Helsinki University Hospital, HUS Diagnostic Center, Haartmaninkatu 3, N00029 HUS, Helsinki, Finland; 2 Faculty of Biological and Environmental Sciences, Molecular and Integrative Biosciences Research Programme, University of Helsinki, Helsinki, Finland; 3 Department of Infectious Diseases, Inflammation Center, Helsinki University Central Hospital, Helsinki, Finland; 4 Clinical Microbiology, Turku University Hospital and University of Turku, Turku, Finland

**Keywords:** *Aerococcus urinae*, Bacteraemic infections, Susceptibility testing, Benzylpenicillin, Cefuroxime, Ceftriaxone

## Abstract

Our objective was to assess the incidence of bacteraemic *Aerococcus urinae* cases at Helsinki metropolitan area, Finland, from a 6-year study period (2013 to 2018) and to further characterize available cases. The study evaluates the outcome of commonly used cefuroxime treatment and determinate a set of *A. urinae* in vitro antimicrobial susceptibilities for benzylpenicillin, cefuroxime, and ceftriaxone. Clinical records of *A. urinae* bacteraemic patients were reviewed retrospectively. Antimicrobial susceptibility testing was performed by disk diffusion, gradient test, and broth microdilution for 139–141 clinical *A. urinae* isolates. Clinical data of 72/77 patients were combined with the in vitro susceptibilities. We found an increasing number of bacteraemic *A. urinae* cases within 6-year study period (*p* = 0.01). The patients were mainly elderly males, and all suffered from underlying conditions. A total of 27.3% of cases (21/77) showed polymicrobial blood cultures. Thirty-day mortality was 22.1%. Cefuroxime was the initial empiric antimicrobial agent given for 66/76 of the patients and treatment outcome was favorable for 20/22 patients who received cefuroxime at least up to day 5. All isolates were susceptible to benzylpenicillin and cefuroxime interpreted by EUCAST breakpoints for *Aerococci* and PK-PD breakpoints, respectively. MIC determinations gave variable results for ceftriaxone, 2.1–2.9% of the isolates were resistant. To conclude, it seems that the number of bacteraemic *Aerococcus urinae* cases is increasing at Helsinki metropolitan area, Finland, reflecting the growing blood culture sampling. Clinical *A. urinae* isolates were susceptible to cefuroxime in vitro. Treatment data indicate that empirical cefuroxime started for possibly urinary tract -derived community-acquired bacteraemia covers *A. urinae*.

## Introduction

*Aerococcus urinae*, a Gram-positive coccus, causes urinary tract infections and invasive infections like bacteraemia and infective endocarditis, especially among the elderly [1–6]. In addition to old age, predisposing factors suggested to be associated with *A. urinae* bacteraemia are male gender and underlying urological conditions including catheterization [1, 2]. As a newcomer and rare pathogen, *A. urinae* seems to be an unfamiliar microorganism to most clinicians and the treatment approaches are variable [1, 7]. In Europe, bacteraemic *A. urinae* infections has been characterized comprehensively only in Sweden [2, 7]. Aging in the human population generates global challenges [8] and the resulting multimorbidity may raise the significance of the emerging pathogens like *A. urinae* in the future. A proper managing of infections caused by *A. urinae* is important also from the antibiotic stewardship perspective. Aerococci are catalase negative and resemble α-hemolytic streptococci when growing on blood agar plate but in Gram staining cells appear in clusters like staphylococci [9]. MALDI-TOF mass spectrometry (MS) techniques have substantially improved correct identification of aerococci in clinical microbiology laboratories and contributed to its emergence as a “novel” agent of urinary tract derived infections [2, 10].

Antimicrobial susceptibility breakpoints or interpretive criteria are needed to define clinical susceptibility or resistance of a microbe to a certain antimicrobial. In vitro susceptibility of *A. urinae* has been studied to some extent but there is variation in the used methods and result interpretations [11, 12]. The European Committee on Antimicrobial Susceptibility Testing (EUCAST) recently released clinical breakpoints for *Aerococcus urinae* against certain penicillins, fluoroquinolones, meropenem, vancomycin, nitrofurantoin, and rifampicin [13]. In Finland, the empiric starting therapy for community-acquired bacteraemias is the second-generation cephalosporin cefuroxime. However, EUCAST standard lacks the clinical breakpoints for aerococci and cephalosporins. The aim of the study was to assess the incidence of bacteraemic infections caused by *A. urinae* in Helsinki metropolitan area during the years 2013–2018 and to characterize these cases by reviewing the medical charts retrospectively. We aimed to estimate the treatment outcome of cefuroxime in managing these infections. Three different susceptibility testing methods, i.e., disk diffusion (EUCAST methodology), gradient test (Etest), and broth microdilution (Sensititre), were compared to study in vitro susceptibilities for benzylpenicillin, cefuroxime, and ceftriaxone of a set of clinical *A. urinae* isolates.

## Materials and methods

### Isolates for susceptibility testing

Our laboratory, HUSLAB (Hospital District of Helsinki and Uusimaa Laboratory Services), serves the Helsinki metropolitan area of approximately 1.7 million inhabitants and the specimen catchment area has remained constant during the study period. For susceptibility testing, available stored (− 70 °C) clinical blood culture *Aerococcus urinae* isolates from the years 2013–2018 (*n* = 81) were retrospectively searched from the laboratory database using the WHONET software and the urine culture isolates (*n* = 60) were collected and stored for this study during 2 months in November–December 2017. Isolates were subcultured on horse blood agar, grown in 5% CO_2_ at 35 ± 1 °C for 16–20 h before species identification using MALDI-TOF MS (VITEK MS MALDI-TOF, bioMérieux, France).

### Antimicrobial susceptibility testing

Susceptibility testing of *A. urinae* isolates for benzylpenicillin, cefuroxime, and ceftriaxone was performed in parallel by three different methods; disk diffusion (Oxoid, Cambridge, UK), gradient tests (Etest, BioMérieux SA, Marcy I’Etoile, France), and broth microdilution (Sensititre, Thermo Fisher Scientific, Pittsburgh, PA.). Disk diffusion tests were performed in duplicate according to EUCAST methodology for fastidious organisms. A McFarland 0.5 inoculum measured by Densimat (Biomerieux, SA, Marcy I’Etoile, France) was used on MH-F plates and incubated at 35 ± 1 °C in 5% CO_2_ for 16–20 h. The concentrations of the antibiotic disks were 1 unit for benzylpenicillin, 30 µg for cefuroxime, and 30 µg for ceftriaxone. Gradient tests were performed according to the manufacturer’s instructions in duplicates. Etest gradients used were 0.002–32 mg/L for benzylpenicillin, 0.016–256 mg/L for cefuroxime, and 0.016–256 mg/L for ceftriaxone. If necessary, isolates were re-incubated up to 40–44 h. For broth microdilution, Sensititre Streptococcus species STP6F plate was used. The ranges of penicillin, cefuroxime, and ceftriaxone were 0.03–4 mg/L, 0.5–4 mg/L, and 0.12–2 mg/L, respectively. One hundred microliters of 0.5 McFarland suspension was transferred to 11 mL of Mueller–Hinton broth with lysed horse blood (Thermo Fisher Scientific) and inoculated onto the STP6F plates by Sensititre AIM™ Automated Inoculation Delivery System and incubated for 24 h at 35 °C. Minimal inhibitory concentration (MIC) values were read using Sensititre Vizion™ Digital MIC Viewing System and the Sensititre SWIN Software with semi-automated read options. *Streptococcus pneumoniae* ATCC 49619 was used as the quality control strain for all examined antimicrobial agents.

### Clinical characterization of bacteraemic patients

Medical charts of 77 patients were available for the study and the underlying diseases, symptoms (at admission and during the clinical course of the disease), signs of sepsis (e.g., low blood pressure, higher respiratory rate, altered mental status), and laboratory and imaging findings were evaluated. The antimicrobial treatment, i.e., agents used and length in days throughout the infection episode, was recorded. Evaluations for metastatic infectious lesions and 30-day mortality were recorded. A patient was considered to respond to the antimicrobial therapy if she/he was feverless and if CRP value had decreased by ≥ 10% within days 5–9. Helsinki University Hospital (HUS) and the City of Helsinki approved this study.

### Statistical analysis

Statistical analysis was performed by SPSS Statistics ver. 25.0 (IBM Co., Armonk, NY, USA). Chi-square test was used to study the incidence between the study years. In general, Independent sample *t*-test was used for constant variables and Mann–Whitney U-test for categorical variables and for small sample sizes. Wilcoxon signed-rank test and Pearson correlation were used to compare gradient test and broth microdilution. Pearson correlation was used to evaluate the association between benzylpenicillin and cefuroxime susceptibility results. A *p*-value of < 0.05 was considered statistically significant.

## Results

### *Aerococcus urinae* bacteraemic patients

#### Incidence and microbiological findings

**Fig. 1:**
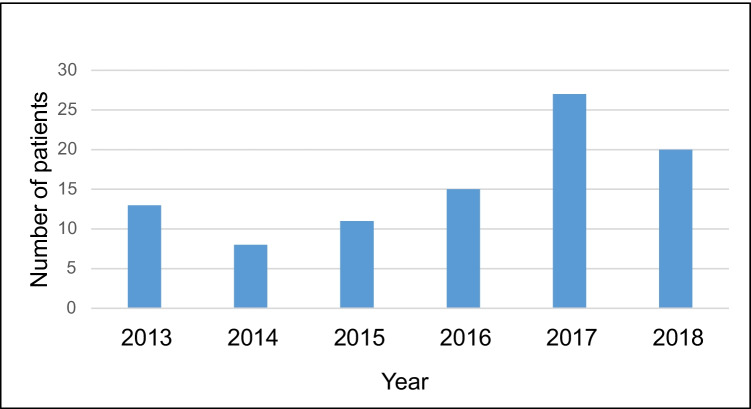
Bacteraemic *A. urinae* cases per study years 2013–2018 (*n* = 94)

During the 6-year study period (years 2013–2018), altogether, 94 patients were diagnosed with bacteraemia caused by *A. urinae* (identified by MALDI-TOF MS) corresponding to incidence of 9.2 cases per 1,000,000 inhabitants per year. Figure 1 represents bacteraemic *A. urinae* cases distributed throughout the study years and the trend seems to be increasing (*p* = 0.01). In the same period, the total amount of taken blood culture samples has also increased steadily from 100,801 blood culture bottles drawn in 2013 to 155,092 bottles drawn in 2018.

Electronic medical charts of 77 (81.9%) patients were accessible and the results given below represent them. Besides *A. urinae*, 27.3% (21/77) of the patients had one or more additional bacterial species in their blood culture samples. Urine culture showed *A. urinae* in 7.8% (6/77), mixed growth in 26.0% (20/77), and other uropathogens in 14.3% (11/77) of the cases. Blood and urine culture findings are described in Table 1.

**Table1:** Blood and urine culture findings of the bacteraemic patients

Culture finding	Number (%)	Bacterial species
From blood		
Pure culture	56/77 (72.7)	*Aerococcus urinae*
*A. urinae* and one additional species	18/77 (23.4)	*Enterococcus faeclis n* = 4
		*Proteus mirabilis*^*a*^* n* = 3
		*Escherichia coli*, *Staphylococcus aureus n* = 2
		*Klebsiella pneumoniae*^*b*^*, Streptococcus anginosus* group*,*
		*Streptococcus agalactiae,* lactobacillus*, Actinotignum schaalii,*
		*Bacteroides fragilis* group*, Clostridium perfringens n* = 1
*A. urinae* and two additional species	3/77 (3.9)	*E. faecalis* and *Staphylococcus epidermidis*
		*Peptinophilus asaccharolyticus* and coagulase negative staphylococcus
		*P. asaccharolyticus* and *A. schaalii*
From urine		
Pure culture	6/77 (7.8)	*A. urinae*
Other uropathogen	11/77 (14.3)	*K. pneumoniae*^*b*^, *P. mirabilis*^*a*^, *E. faecium n* = 3
		*E. coli*, *Klebsiella oxytoca*, *Enterobacter cloacae*, *S. agalactiae n* = 1
Mixed growth^c^	20/77 (26.0)	-
Negative (no growth)	6/77 (7.8)	-
Other status^d^	34/77 (44.2)	-

^a^Both in blood and urine in two cases

^b^Both in blood and urine in one case

^c^ > 2 species without any predominant primary urinary tract pathogen

^d^Urine culture not taken or taken after antimicrobial started

#### Patient characteristics and symptoms

Table 2 defines the clinical characteristics of the bacteraemic patients. They were mainly elderly and male. All patients suffered from one or multiple underlying conditions. Vast majority had cardiovascular disease and three-fourths some underlying urologic or nephrologic disorder. Fever and urinary tract symptoms were common. One-fifth of the patients died within 30 days and they were significantly older compared to the patients who survived (86 years versus 78 years, respectively, *p* = 0.018). Neither group had polymicrobial bacteremia more often (*p* = 0.147).

**Table 2:** Characteristics of the bacteraemic patients including demographics, underlying diseases, the initial signs and symptoms, and other features

Characteristics	No. of patients (*n* = 77)
Demographics	
Mean age in years (range)	80 (47 − 98)
Male gender	61 (79.2%)
Underlying disease^a^	
Patients with cardiovascular diseases	65 (84.4%)
Hypertension	48 (62.3%)
Atrial fibrillation	26 (33.8%)
Ischemic heart disease	17 (22.1%)
Patients with urologic or nephrologic disorder	57 (74.0%)
Benign prostatic hypertrophy	25 (41.0%)
Prostatic cancer	13 (21.3%)
Indwelling urinary catheter	17 (22.1%)
Urinary tract strictures or stone formation	18 (23.4%)
Patients with neurologic disorder	51 (66.2%)
Memory disorder	28 (36.4%)
Cerebrovascular disorder	23 (29.9%)
Patients with other malignancy (current or past)	13 (16.9%)
Patients with diabetes	16 (20.8%)
Signs and symptoms (at hospital arrival) ^a^	
Fever (≥ 38 °C) ^c^	63 (81.8%)
Urinary tract symptoms	45 (58.4%)
Functional decline	31 (40.3%)
Cognitive decline	15 (19.5%)
Gastrointestinal symptoms	21 (27.3%)
Other symptoms	
Cardiac symptoms	11 (14.3%)
Articular symptoms	5 (6.5%)
Other	
30-day mortality	17 (22.1%)
Mean duration of hospitalization in days (range)	20 (0 − 126)
Intensive care unit (ICU) admission	7 (9.1%)
Mean duration of ICU in days (range)	6 (1 − 15)
Institutional care	21 (27.3%)
Penicillin allergy	3 (4.1%) ^d^

^a^No significant difference between died and survived patients (data not shown)

^b^Percentage of men (*n* = 61)

^c^Mentioned in the preliminary report or measured during hospitalization

^d^Number of patients *n* = 73

#### Clinical findings during disease

Table 3 shows the initial clinical presentations and the follow-up data. Most patients presented with fever and increased inflammatory markers at the onset of infection. Both CRP and total white cell counts decreased by days 5–9 and only four patients still had fever. Notably, the patients who died had significantly higher CRP within days 5–9 compared to the patients who survived (96 mg/L versus 43 mg/L, respectively, *p* = 0.003).

**Table 3:** Clinical and laboratory findings of the bacteraemic patients. Initial finding (Day 0) refers to the first parameter measured after hospitalization

Finding	Initial = day 0	Days 0 − 4	Days 5 − 9
Mean temperature °C (range)	38.0 (33.2 − 40.0)	-	-
Fever ≥ 38 °C	45/77 (58.4%)	-	4/73 (5.5%)
Mean CRP, mg/L (range)	94 (< 3 − 416)	185 (25 − 466)	53 (< 3 − 278)^a^
Mean leucocyte, E9/L (range)	12.2 (1.3 − 40.2)	17.3 (2.0 − 40.8)	9.4 (4.5 − 22.4)^a^
Kidney insufficiency GFR^b^ < 60^c^	39/75 (52.0%)	-	-
Hypotension < 100 mmHg systolic	11/76 (14.5%)	-	-
Response to therapy^d^	-	-	60/65 (92.3%)^e^

^a^Statistically significantly higher in patients who died within 30 days

^b^*GFR*, glomerular filtration rate

^c^Women had kidney insufficiency more often than men (86% versus 44%, respectively, *p* = 0.005)

^d^No fever and CRP decreased ≥ 10%

^e^Complete data (CRP value or temperature °C) was missing from 12 patients

The following radiological examinations were performed: abdominal/urinary tract computed tomography (CT) for 13/77 (16.9%) of the patients, body CT scan for 7/77 (9.1%), chest CT scan for 2/77 (2.6), abdominal ultrasound for 17/77 (22.1%), urinary tract ultrasound for 6/77 (7.8%), and transthoracic/transesophageal echocardiography for 17/77 (22.1%). Metastatic infectious lesions were rare. Two patients (2.6%) had diagnosed endocarditis, one patient (1.3%) suffered from spondylodiscitis, and three patients (3.9%) had abscesses (1 kidney, 1 paravertebral abscess, and 1 subcutaneal abscess in the lower abdominal wall). In addition, one patient had *S. aureus* abscess in an ankle simultaneously with the *A. urinae* bacteremia. Most patients did not undergo any radiological examinations to search for metastatic infectious lesions.

#### Antimicrobial therapy

Table 4 summarizes the used antimicrobial agents. One patient apparently did not receive any antimicrobial treatment (and survived the infection). In initial therapy, eight different antimicrobials or their combinations were used: cefuroxime, cefuroxime combined with tobramycin, cefuroxime combined with metronidazole, ceftriaxone, amoxicillin, cloxacillin, piperacillin-tazobactam, or ciprofloxacin. Cefuroxime was the initial empiric antimicrobial given for majority of the patients (86.8%, 66/76) with a typical dose of 1.5 g three times daily intravenously. Cefuroxime was equally used as the initial drug among patients who died (82.4%, 14/17) and survived (88.1%, 52/59) (*p* = 0.537). The mean length of the initial cefuroxime treatment was 4 days (range 1–11). On day 5 after hospitalization, cefuroxime was given for 28.9% (22/76) of the patients, of which majority (90.9%, 20/22) survived. There were no differences in cefuroxime MIC values between dead and survivors (*p* = 0.339 for gradient tests; *p* = 0.100 for broth microdilution). Antimicrobial de-escalation from the first empirical choice to benzylpenicillin, ampicillin, or amoxicillin (with or without clavulanic acid) during days 1–5 was introduced among 30.3% (23/76) of the cases. In the remaining cases (69.7%, 53/76), the choice of the second, third, and further antimicrobial agents seemed rather random.

**Table 4:** Antimicrobial treatment of bacteraemic patients (*n* = 77)

Antimicrobial agent	Initial agent (*n* = 76)	2nd agent (*n* = 70)	3rd agent (*n* = 45)	4th agent (*n* = 21)	5th agent (*n* = 6)
Cefuroxime	66 (86.8%)	1 (1.4%)	5 (11.1%)	-	1 (16.7%)
Cefuroxime + other agenta	3 (3.9%)	5 (7.1%)	1 (2.2%)	1 (4.8%)	-
Ceftriaxone	2 (2.6%)	4 (5.7%)	4 (8.9%)	4 (14.3%)b	-
Cefalexin	-	3 (4.3%)	1 (2.2%)c	1 (4.8%)	-
Penicillins	2 (2.6%)	34 (48.6%)	22 (48.9%)	9 (42.9%)	3 (50%)
Benzylpenicillin	-	11 (15.7%)	4 (8.9%)	4 (19.0%)^d^	-
Phenoxymethylpenicillin	-	-	4 (8.9%)	1 (4.8%)	2 (33.3%)^d^
Ampicillin	-	7 (10.0%)^d^	1 (2.2%)	-	-
Amoxicillin	1 (1.3%)	7 (10.0%)	12 (26.7%)^c^	2 (9.5%)	1 (16.7%)
Amoxicillin with clavulanic acid	-	5 (7.1%)	1 (2.2%)	2 (9.5%)	-
Cloxacillin	1 (1.3%)	4 (5.7%)^e^	-	-	
Broad-spectrum^f^	4 (5.3%)	11 (15.7%)	5 (11.1%)^d^	2 (9.5%)	2 (33.3%)
Other^g^	1 (1.3%)	12 (17.1%)	7 (15.6%)^h^	4 (19.0%)	-

^a^Other agent = clindamycin, metronidazole, tobramycin, moxifloxacin, and vancomycin

^b^Three cases combined with (1) clindamycin (2) piperacillin-tazobactam and (3) linezolid

^c^One case combined with metronidazole

^d^One case combined with levofloxacin

^e^One case combined with vancomycin

^f^Imipenem, meropenem, and piperacillin-tazobactam

^g^Ciprofloxacin, clindamycin, gentamicin, levofloxacin, nitrofurantoin, sulfa-trimethoprim, and vancomycin

^h^In one case levofloxacin combined with vancomycin

### Antimicrobial susceptibilities

**Fig. 2:**
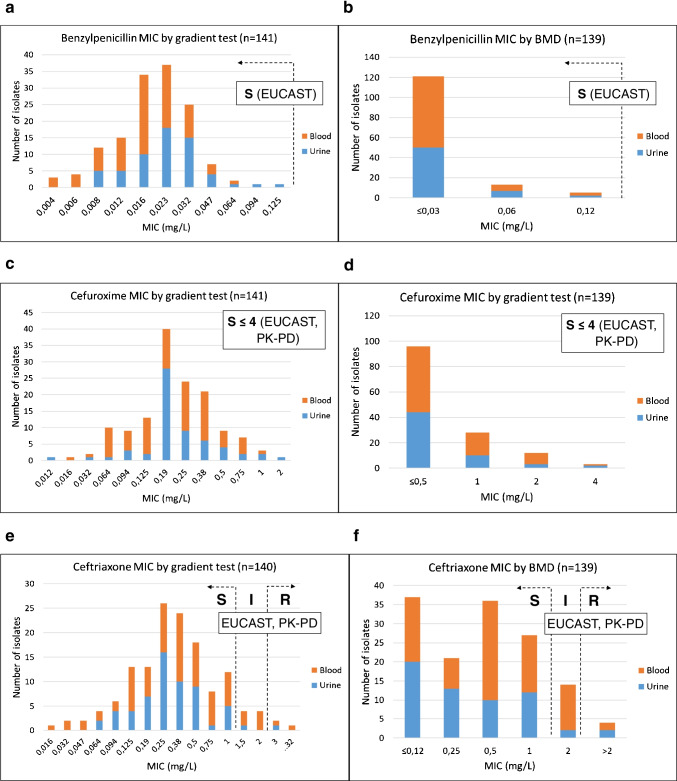
Minimal inhibitory concentrations (MICs) against *A. urinae* isolates performed by gradient diffusion test and broth microdilution method (BMD). **a** Benzylpenicillin MICs by gradient diffusion. **b** Benzylpenicillin MICs by BMD. **c** Cefuroxime MICs by gradient diffusion. **d** Cefuroxime MICs by BMD. **e** Ceftriaxone MICs by gradient diffusion. **f** Ceftriaxone MICs by BMD. For penicillin, EUCAST breakpoints for *Aerococci* were used to interpret the results. For cefuroxime and ceftriaxone, EUCAST PK-PD breakpoints were used. *S*, susceptible; *I*, susceptible; increased exposure; *R*, resistant; *PK-PD*, pharmacokinetic-pharmacodynamic

Figure 2 shows that MICs of blood and urine isolates distributed quite equally, and all studied isolates were susceptible (S) to benzylpenicillin and cefuroxime. According to gradient tests, nearly all isolates (97.9%, 137/140) were susceptible to ceftriaxone but 5.7% (8/140) were susceptible with increased exposure (I). The remaining 2.1% (3/140) of the isolates were resistant (R) to ceftriaxone. Broth microdilution gave rather similar percentages for ceftriaxone: 97.1% (135/139) of the isolates were S, 10.1% (14/139) were I, and 2.9% (4/139) were R. Benzylpenicillin MICs were interpreted according to EUCAST *A. urinae* breakpoints (S ≤ 0.125, R > 0.125), while EUCAST PK-PD breakpoints were used to interpret cefuroxime (S ≤ 4, R > 8) and ceftriaxone (S ≤ 1, R > 2) MICs.

Table 5 describes the susceptibility results performed by disk diffusion method and compares the results obtained from MIC determinations. MIC values performed by gradient test and broth microdilution correlated statistically significantly among all tested antimicrobial agents. However, broth microdilution gave on average higher MIC values, even when gradient test values were converted to correspond to broth microdilution values (analyzed by Wilcoxon signed-rank test, data not shown). There was also discrepancy among ceftriaxone MIC interpretations. Two out of 139 isolates (1.4%) were R by broth microdilution but S or I by gradient test (very major errors). Vice versa, 1/139 isolate (0.7%) was R by gradient test but S by broth microdilution (a major error). According to disk diffusion, all isolates were susceptible to benzylpenicillin (S ≥ 21 mm) interpreted using EUCAST *A. urinae* breakpoints. Penicillin and cefuroxime zone diameters correlated significantly (Pearson correlation 0.924, *p* < 0.001).

**Table 5:** The effects of the antimicrobial agents benzylpenicillin, cefuroxime and ceftriaxone against 139–141 *Aerococcus urinae* isolates. Zone diameters (mm), MIC50, MIC90, and MIC ranges are described. The correlation and the number of susceptibility interpretations (S/I/R) between MIC methods are shown

Antimicrobial agent	Disk diffusion mm	Gradient test				Broth microdilution (BMD)				Gradient vs. BMD
		MIC (mg/L)				MIC (mg/L)				Pearson correlation
		MIC_50_^a^	MIC_90_^b^	Range	S/I/R^c^	MIC_50_^a^	MIC_90_^b^	Range	S/I/R^c^	
Benzylpenicillin	26–47	0.023	0.032	0.004–0.125	141/0/0	≤ 0.03	0.06	≤ 0.03–0.12	139/0/0	0.428, *p* < 0.001
Cefuroxime	24–47	0.19	0.5	0.012–2	141/0/0	0.5	2	0.5–4.0	139/0/0	0.622, *p* < 0.001
Ceftriaxone	23–47	0.38	1.0	0.016–32	129/8/3	0.5	2	0.12– > 2	121/14/4	0.579, *p* < 0.001

^a^MIC value at which ≥ 50% of the isolates are inhibited

^b^MIC value at which ≥ 90% of the isolates are inhibited

^c^*S*, susceptible; *I*, susceptible, increased exposure; *R*, resistant

## Discussion

We found an increasing number of bacteraemic *Aerococcus urinae* cases at Helsinki Metropolitan area, Finland. This may reflect the increase in total amount of blood culture samples drawn. Nevertheless, this observation is worth noticing as aging in human population may rise the significance of *A. urinae* as a causative organism in blood stream infections among elderly. Further surveillance is needed to follow up the incidence.

Consistently with previous studies [1, 2], we found that bacteraemic patients with *A. urinae* were mainly elderly men with several comorbidities. *A. urinae* was found only in a small fraction of the urine samples although urogenital tract was the probable infection focus on many cases, as in previously published data [7]. Mixed flora reported in urine cultures may have covered possible underlying *A. urinae* growth, which calls for the importance of proper urine sampling techniques and experience. However, also nearly one-third of bacteremic cases showed polymicrobial blood culture findings. It may be possible that *A. urinae* in blood culture originates from another source than the urinary tract, e.g., from the upper respiratory tract or from the gastro-intestinal system. In surprisingly many cases, urine sample was taken only after antimicrobial therapy had been initiated or not at all. Metastatic infectious lesions were found only seldom but notably deep focuses were not systemically searched for in all patients due to their poor condition or old age.

In our cohort, the 22% mortality rate was somewhat higher than reported previously [1, 2, 4, 7, 14]. In other studies, 30-day mortality has ranged between 6 and 17%. We found that patients who died were on average older compared to survivors. Patients responded to the given antimicrobial treatment mostly well based on declined fever and CRP. However, CRP declined significantly slower for patients who died. Mortality was not enhanced by polymicrobial finding in blood culture. As far as we know, cefuroxime treatment for bacteraemic *A. urinae* infections has not been reported before. Cefuroxime was the first empiric antimicrobial choice in most of the cases and based on our data, we did not find evidence against its use. Rasmussen [9] states in his review article that the use of cephalosporins in *A. urinae* infections lack rationale due to worse pharmacodynamics apparently comparing to penicillin. Nevertheless, we suppose that empirical cefuroxime treatment for a suspected urinary tract–derived community-acquired bacteraemia also covers *A. urinae*, a rare causative agent in bacteraemic infections. De-escalation to benzylpenicillin, ampicillin, or amoxicillin should be performed when *A. urinae* is recognized by name in blood culture report and penicillin allergy or mixed infection is not a concern.

All tested *A. urinae* isolates were susceptible to cefuroxime (MIC ≤ 4 mg/L, using EUCAST PK-PD breakpoints) and clinical response was not inferior compared to other agents used in the studied patient cohort. In accordance with the previous findings [7, 15], all isolates were susceptible to benzylpenicillin. Benzylpenicillin and cefuroxime zone diameters correlated well, suggesting that susceptibility to cefuroxime could be interpreted from benzylpenicillin zone millimeters. On the contrary, the ceftriaxone MIC distribution showed that 2.1–2.9% of the isolates were resistant to ceftriaxone (MIC > 2 mg/L). In our study, ceftriaxone was used only for few patients, so we were not able to analyze the treatment outcome of the agent. Ceftriaxone susceptibility may need separate testing, and not interpretation from benzylpenicillin susceptibility. Ceftriaxone resistance among *A. urinae* has been described previously [5, 11, 16, 17] but its clinical relevance has not yet been resolved. The proportion of resistant isolates depends on the interpretation criteria. When EUCAST breakpoints for viridans group streptococci (≤ 0.5 mg/L) were used, 76% of the studied *A. urinae* isolates were considered susceptible [9]. In our material, 107/139 (77%) of the studied isolates had ceftriaxone MIC value ≤ 0.5 mg/L. Finnish *A. urinae* isolates resemble those studied in other countries when concerning benzylpenicillin and ceftriaxone in vitro susceptibility.

Carkaci and co-workers [15] reported parallel susceptibility testing on *A. urinae* isolates by gradient test and broth microdilution and they found that penicillin and cefotaxime MICs were in accordance within one dilution. In our study, broth microdilution gave somewhat higher MIC values compared to gradient test for all studied antimicrobials. Previous studies have reported inconsistent MIC values obtained by Etest versus broth microdilution, e.g., for fluoroquinolones [18] and for tetracycline and erythromycin [19]. Clinically most serious implications result when true-resistant isolate is categorized as susceptible leading likely to therapeutic failure [20]. Ceftriaxone MIC interpretations from our data resulted in two very major errors and one major error. Cantón and colleagues [21] determined *Staphylococcus aureus* ceftaroline MICs and found that isolates were more often ceftaroline resistant by broth microdilution as compared to Etest. In addition, discrepancy observed with *A. urinae* clindamycin susceptibility indicates that broth microdilution gives more resistant results than Etest [9]. Precaution should be followed when performing and interpreting ceftriaxone susceptibility to *A. urinae*.

The β-lactam antibiotics inhibit penicillin-binding proteins (PBPs) which are essential for peptidoglycan biosynthesis of bacteria. Acquired or endogenous alterations in PBPs are related to β-lactam resistance [22]. Little is known about PBPs of aerococci. Oskooi et al. have reported differences in MIC-values between certain cephalosporins, namely cefalotin (range 0.023–0.5) and ceftibuten (> 32) in aerococci [23]. Ceftriaxone non-susceptible *A. urinae* findings indicate that alterations in PBPs may have occurred. It is possible that different PBPs are related to the mechanism of action of benzylpenicillin versus ceftriaxone as the same isolate can show susceptibility to benzylpenicillin but resistance to ceftriaxone. Research concerning PBPs of aerococci is needed to understand the probable underlying mechanisms.

The strengths of our study include relatively large study population representing 141 studied *A. urinae* isolates from two distinct body sites. In addition, three parallel in vitro susceptibility methods were used, two of which were performed as duplicates. Species identification method used (MALDI-TOF MS) is well validated and was consistent during the study years. The major limitation is the retrospective nature of the study design. The documentation of the medical records was incomplete in many cases. For susceptibility analysis, geographic area is limited. *A. urinae* bacteremia was considered as a factor contributing to death in most of the cases. However, all patients had comorbidities and a majority were elderly. Conclusions about treatment outcomes are inevitably simplifying.

To conclude, *Aerococcus urinae* bacteraemias appear to increase at Helsinki Metropolitan area, Finland, and it seems that clinicians are unfamiliar with this pathogen and the optimal treatment of bacteraemic aerococcal infections. Based on our findings, cefuroxime is a possible choice for empiric treatment to cover *A. urinae* bacteraemia. Almost one-third of bacteraemic cases were polymicrobial and cefuroxime covered most of these findings. In bacteraemic cases caused by *A. urinae* alone, definitive penicillin therapy should be considered. Our study complements the understanding about bacteraemic infections caused by *Aerococcus urinae* and introduces a set of cefuroxime in vitro susceptibility data for this species.

## Data Availability

Not applicable.
